# Tobacco Use and Cessation among a Nationally Representative Sample of Men in India, 2019–2021

**DOI:** 10.1155/2023/4292647

**Published:** 2023-03-22

**Authors:** S. K. Singh, Shubham Kumar, Gyan Chandra Kashyap

**Affiliations:** ^1^Department of Survey Research and Data Analytics, International Institute for Population Sciences, Govandi Station Road, Deonar, 400088, Mumbai, India; ^2^Institute of Health Management Research (IIHMR), 560100, Bangalore, India

## Abstract

Tobacco users are exposed to a higher risk of noncommunicable diseases, leading to premature mortality and disability-adjusted life years (DALYs). The future prediction indicates that tobacco-related mortality and morbidity rates will substantially increase in coming years. The study is aimed at assessing the prevalence of tobacco consumption and cessation attempts for different tobacco products among adult men in India. The study utilized information from India's latest National Family Health Survey-5 (NFHS-5) data which was conducted during 2019-21, including 988,713 adult men aged 15 years and above and 93,144 men aged 15-49. Results suggest that 38 percent of men consume tobacco, including 29% in urban and 43% in rural areas. Among the men aged 35-49 years, the odds were significantly higher for consuming any form of tobacco (AOR: 7.36, CI: 6.72-8.05), smoking cigarettes (AOR: 2.56, CI: 2.23-2.94), and smoking bidi (AOR: 7.12, CI: 4.75-8.82) as compared to those aged 15-19. The application of multilevel model indicates that tobacco usages are not evenly distributed. In addition, there is maximum clustering of tobacco usages found around household level factors. Further, 30% of men aged 35-49 years attempted to stop consuming tobacco. Though 27% of men tried to quit tobacco in the last 12 months and 69% of men are exposed to secondhand smoke, 51% of men who received advice for quitting tobacco and visited the hospital in the last 12 months belong to the lowest wealth quintile. These findings prioritize promoting awareness about adverse effects of tobacco use, especially in rural areas, and capacitate them to adopt cessation efforts so that those who want to quit may be successful in their efforts. In addition, the health system's response to the tobacco epidemic in the country should be strengthened by training of service providers to promote cessation efforts through appropriate counselling of all the patients visiting them in the context of tobacco use in any form as key drivers of the increasing burden of noncommunicable diseases (NCDs) in the country.

## 1. Introduction

Tobacco users are constantly exposed to the risk of noncommunicable diseases, leading to premature mortality among the risk population. Globally, eight million deaths are reported every year due to tobacco use [1]. Consequently, smokeless tobacco and cigarette smoking contribute to six and five percent disability-adjusted life years (DALYs), respectively [2]. However, the prevalence of tobacco use has been decreasing globally since 1990 [2], and most tobacco users live in low-middle-income countries (LMICs) [3]. India is also no exception compared with other LMICs; for example, Global Adult Tobacco Survey-2 (GATS) highlighted that nearly 29 percent of adults (42% male and 14% female) aged 15 years and above use some form of tobacco [4]. In addition, the fourth round of the National Family Health Survey (NFHS-4), conducted in 2015-16, showed that approximately 46% of males were tobacco users [5]. Consequently, one million deaths were reported in India, and one-sixth of the world's deaths were due to tobacco use [6]. The future prediction indicates that tobacco-related mortality and morbidity rates will substantially increase by 2050 and beyond [7].

Various tobacco products are available, which can be smoked, such as cigarettes, or chewed, such as different smokeless tobacco products. Furthermore, smoked tobacco includes cigars, cigarettes, bidis, hookah, pipes, and chillum, while smokeless tobacco includes khaini, gutka, pan with tobacco, and betel nut. Cigarettes are the most commonly consumable tobacco product worldwide [8]. In the Indian scenario, 2015-16, NFHS data revealed that cigarette smoking (32%) was dominant among men tobacco users, followed by chewing tobacco (2%) and cigar (0.23%). In addition, thirty percent of men use at least one tobacco product, eight percent are dual tobacco users, and two percent are polytobacco users [9].

Considering the socioecological differentials in tobacco products, including individual-level socioeconomic and contextual determinants, place of living is always concerned in terms of disparity in health issues due to cultural lag. People residing in rural areas are always disadvantaged regarding education, health awareness, healthcare utilization, etc. Extensive literature is available on rural-urban differentials in the use of tobacco products [10–12]. The substantial rural-urban gap in the prevalence of current tobacco use was highlighted in a study conducted in India by Singh and Ladusingh that showed 52% prevalence in rural arears compared to 38% in urban ones [13].

Many countries have adopted different policies to reduce tobacco use. However, quitting tobacco is quite a complex process, although some studies have tried to emphasize this issue [14–16]. Healthcare providers are the key player in the cessation of tobacco use. With understanding of the globalization of the tobacco epidemic, the World Health Organization (WHO) took the initiative framework convention on tobacco control (WHO, FCTC) which was the first international treaty on tobacco control, participated by 181 countries held in 2003 [17]. The prime objective of the convention was to reduce the demand and supply of tobacco products, to control tobacco use. In this regard, the WHO introduced MPOWER measures, which is an effective measure to prevent tobacco use. The MPOWER packages provide six essential parameters to control the tobacco epidemic. In addition to that, it (MPOWER) indicates the following: M stands for monitor tobacco use, P stands for protecting people from tobacco smoke, O stands for offering help to quit, W stands for warning about the dangers of tobacco, E stands for enforcing bans on tobacco advertising, promotion, and sponsorship, and R stands for raising taxes on tobacco products [18]. GATS data provide comprehensive measures of MPOWER in the Indian scenario. In addition, a study highlighted that concrete action still needs to be addressed if the noncommunicable disease global voluntary target of a 30% reduction by 2025 in the prevalence of tobacco use among the 15 years and above population is to be achieved [19].

India is also not behind legislating tobacco policies and programs to control the tobacco epidemic in the country. The government of India came up with the cigarette act in 1975 to regulate the production, supply, and distribution of smoking tobacco [20]. Further, one major decision was taken in 1992, as tobacco in all dental products was banned [21]. Furthermore, a significant act was introduced in 2003 called the Cigarettes and Other Tobacco Products Act (COTPA) with the prohibition of advertisement and regulation of trade and commerce, production, supply, and distribution of tobacco products in India [22]. In addition, the act provides the provision restriction of smoking in public places, prohibition of advertisements of tobacco products, prohibition of the sale of tobacco products to minors aged below 18 years, ban on the sale of tobacco products within 100 yards of educational institutions, and mandatory display of pictorial health warning on the tobacco products. An evolutional study for the COTPA act was carried out in 2019 for the urban area among educational institutions and points of tobacco sales. The study revealed that, overall, complying with the norms of the COTPA act in urban areas, especially in Shimla, no tobacco-selling vendors were found selling tobacco to minors [23].

Even though several research studies have been done on tobacco consumption concerning different characteristics such as age, gender, place of residence, and media exposure, only a few studies had explored the current status of tobacco consumption with their cessation efforts made in the Indian context [13, 24]. With the increasing awareness and various other interventions, the variety of tobacco shows a crest and trough in consumption status. This study estimates the prevalence of a variety of tobacco concerning their consumption and cessation attempts among adults in India.

Consequently, there was a reduced roll tobacco use among adult men, although some socioecological and contextual factors still affect tobacco use in rural and urban areas among men. So, with the latest demographic and health survey data, this paper analyzes the prevalence, determinants, and cessation of tobacco use among men in India and focuses at the key strategies to address their dilemmas of increased use vis-a-vis cessation in various contexts.

## 2. Material and Methods

### 2.1. Data Source

The study utilized information from India's latest National Family Health Survey-5 (NFHS-5) data. The NFHS is a nationally represented district-level survey conducted for all 36 states and union territories (UTs). The nodal agency for conducting this survey is the International Institute for Population Sciences (IIPS), Mumbai. The survey is cross-sectional and adopted a systematic, two-stage cluster sample of the household. The NFHS survey provides comprehensive information on several emerging issues, including family planning, education, nutrition, health problems, and health-related behaviors. This study includes 988,713 adult men aged 15 years and above, including 93,144 men aged 15-49. In addition, all the men's interviews were done in the households selected for the state module among those aged 15 to 49. Additional information was collected from men 15 years and above at the district level.

### 2.2. Dependent Variables

The dependent variable for this study is current tobacco use by adult men in India. The response was recorded by asking the question “does currently smoke or use tobacco in any form?” The response was recorded as either “yes” or “no” where “no” is considered as a reference category in the analysis Further, to estimate the prevalence of tobacco use by adults by selected sociodemographic parameters, we used “cigarette smoking,” “bidi smoking,” and “smoke five or more cigarette/bidi” as dependent variables. Moreover, to estimate the prevalence of adults who had tried to quit smoking, we used “tried to stop smoking or to use tobacco in any other form in the past 12 months” and “advised to quit smoking or using tobacco in any form” among those who visited a doctor or other healthcare provider in the past 12 months as dependent variables. Lastly, we also used secondhand smoke as a dependent variable by asking “does anyone present when someone (other than themselves) was smoking in the home or elsewhere in the past 30 days?”

### 2.3. Covariates

The covariates for this study are age (15-19, 20-34, 35-49, 50-64, and 60 and above), level of schooling (no schooling, <5 years complete, 5-7 years complete, 8-9 years complete, 10-11 years complete, and 12 or more years complete), the religion of the household head (Hindu, Muslim, and others), caste/tribe of the household head (scheduled caste, scheduled tribe, other backward class, and others), and wealth quintile (lowest, second, middle, fourth, and highest).

### 2.4. Statistical Measures

The bivariate analysis was used to estimate the prevalence of tobacco use, quitting tobacco, and secondhand smoke associated with selected background variables and states/UTs in India. A multivariate logistic regression model was adopted to estimate the adjusted effect of various socioeconomic factors on different types of tobacco use (any tobacco use, smoking cigarette, and smoking bidi). The equation for multivariate logistic regression is given below.

(1) $$\begin{array}{c} \log\left(\frac{p_{i}}{1-p_{i}}\right)=\text{logit}\left(p_{i}\right)=\beta_{0}+\beta_{1}x_{1}+\beta_{2}x_{2}+\cdots +\beta_{n}x_{n}. \end{array}$$

The above regression equation portrays the log odds of probability of consuming any type of tobacco or smoking various tobacco products under the effect of various socioeconomic and behavioral drivers of tobacco use/smoking, i.e., *x*_1_, *x*_2_, *x*_3_, ⋯⋯⋯*x*_*n*_. In addition, to check the association between dependent and independent variables, a chi-square test has been performed.

Further, to see the extent of clustering in tobacco consumption at various levels, we have used multilevel random and fixed effect binary logistic regression model and calculated the ICC values. A multilevel logistic regression model with a random intercept was used to understand clustering in any tobacco use within PSU or “community” and households [25]. The application of the multilevel modeling was justified by the hierarchal structure of the survey, where men were nested within household and the household were nested within PSUs. In a preliminary analysis, a “baseline” or intercept model was examined only to assess the extent of the dependent variable's variation between “communities” and the advisability of using a multilevel modelling strategy. In multilevel analysis, a systematic model building procedure was adopted to finalize the key covariates to be included in the model. Finally, the model included sociodemographic and economic variables such as respondents' age, educational attainments of the respondents, place of residence, caste affiliation, household wealth, and the religious affiliation of the respondents The following is the equation of the model:

(2) $$\begin{array}{c} \log\left(\frac{\pi_{ijk}}{1-\pi_{ijkl}}\right)=Y_{ijkl}=\alpha =\beta X_{ijkl}+\gamma Z_{jkl}+\delta W_{kl}+\varphi U_{l}+r_{0l}+s_{0kl}+d_{0jkl}+e_{0ijkl}, \end{array}$$

where *Y*_*ijk*_ is the current tobacco consumption for individual *i* in the household *j* in PSU *k* in state *l*. In addition, *α* = constant, *X*_*ijkl*_, *Z*_*jkl*_, *W*_*kl*_, and *U*_*l*_ are the vectors of variable; *β*, *γ*, *δ*, and *φ* are the regression coefficients; and *r*_0*l*_, *s*_0*kl*_, *d*_0*jkl*_, and *e*_0*ijkl*_ are the residuals at the individual level, state level, PSU level, and household level, respectively.

The outcomes of the multilevel mixed effect logit regression have been done at 95% confidence interval to show fixed effect of the explanatory variables. Further, to show the random effect, intraclass correlation coefficient (ICC) was computed at the state level, PSU level, and household level. The following is the equation of the ICC:

(3) $$\begin{array}{c} \text{ICC}=\frac{{\text{VAR}}_{n}}{\langle \sum_{n=2}^{N}{\text{VAR}}_{n}+\left(\pi^{2}/3\right)\rangle }, \end{array}$$

where ICC is the intraclass correlation coefficient and VAR_*n*_ is the variance at the *n*th level of regression.

All the analyses were performed using STATA version 17 software. Further, map was drawn using GIS version 10.2.

## 3. Results

The prevalence of tobacco consumption among men aged 15 and above who live in rural and urban areas is presented in Table 1. The study includes 988,788 men aged 15 and above years. The overall prevalence of tobacco consumption (any form) was 38%, which varies from 29% in urban areas to 43% among men living in rural areas. Tobacco uses are more prevalent in rural areas than in urban areas. The prevalence of tobacco use increased along with age; the prevalence is the highest in the age category 50-64 years across rural and urban areas. Men without schooling had the highest prevalence (65%) of tobacco use in rural areas. The prevalence shows a declining trend as the level of schooling increases in rural and urban areas. Men who belong to the schedule tribe category and reside in rural areas had the highest prevalence of tobacco consumption (53%). Also, the predictor wealth quintile shows a similar pattern. Men from the lowest wealth quintile had the highest prevalence of tobacco consumption (58%), while it was the lowest among men belonging to the highest wealth quintile (21%).

**Figure 1:**
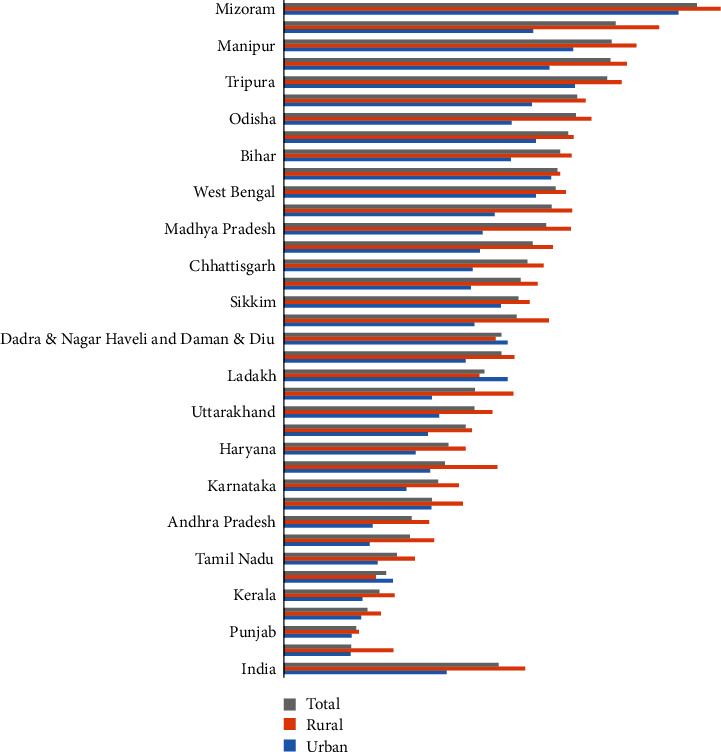
Percentage of the men age 15 years and above who currently use any tobacco by urban, rural, and total.

Figure 1 shows the prevalence of tobacco consumption among men aged 15 and above by their place of residence (rural, urban, and total) across the states/UTs in India. Among all Indian states and union territories (UTs), tobacco consumption was the highest in northeastern states, such as Mizoram (73%) has the highest percentage of tobacco users of any state/UT, followed by Andaman and Nicobar (59%), Manipur and Meghalaya (58% each), Tripura (57%), Assam and Odisha (52%), and Arunachal Pradesh (50%). In addition, Chandigarh, Punjab, Puducherry, Kerala, and Goa have the lowest tobacco use prevalence (less than 20%).

**Figure 2:**
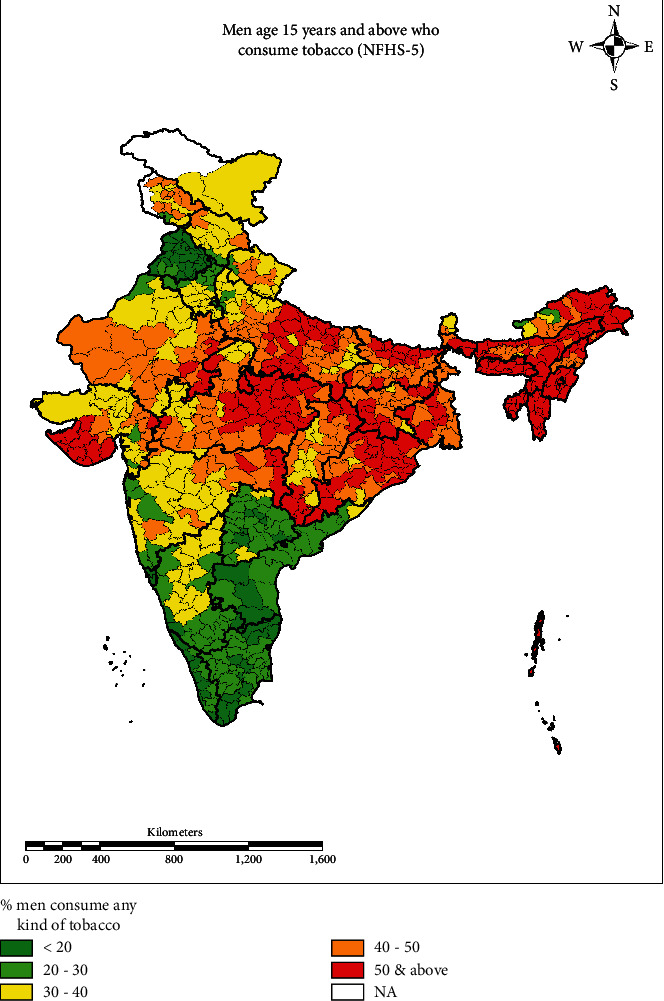
Proportion of men with tobacco consumption age 15 years and above at the district level.

District-level prevalence of tobacco consumption (any form of tobacco) among men aged 15 and above years shown in Figure 2. Results show that most of the districts of southern states, such as Telangana, Andhra Pradesh, and Tamil Nadu, found a lower prevalence of consumption (less than 20%). Moreover, northeastern states reported higher tobacco consumption (50% and above) in almost all the districts. The higher prevalence (50% and above) of tobacco consumption was reported in almost all the districts of Mizoram, Assam, Meghalaya, and Arunachal Pradesh.

Table 2 describes tobacco smoking in different forms by men aged 15-49 and 15-54 years in rural and urban India. From the result, cigarette smoking (13%) and bidi smoking (7%) were more frequent than other tobacco products, such as paan masala or gutka, khaini, and bidis, in rural and urban areas. Bidi smoking (8.3%) and the use of khaini (14.5%) were greater in rural areas than in urban areas. Around seven percent of cigarette smokers from urban areas smoke 10-14 cigarettes per day, and 18% smoke 5-9 cigarettes daily. Similarly, a significant proportion of the men (21%) who live in rural areas smoked 15-24 bidis per day. Moreover, among tobacco users, 50% smoke an average of less than five bidi/cigarette per day in rural areas while 56% in urban areas. Also, 15% of men use an average of 10-14 bidi/cigarette per day among 15-49 years, and at the same time, there was not much difference in the tobacco smoking in the age category 15-49 and 15-54 years.

The prevalence of any kind of tobacco use, smoking cigarette, and bidi five or more per day by their background characteristics is presented in Table 3. The consumption of any form of tobacco and smoking five or more cigarettes/bidis was the highest in the 35-49 years of age group (51% and 57%, respectively) than the smokers of other age categories. Results found a significant difference (10%) among the users of tobacco who consume any kind of tobacco live in rural areas than the urban areas. According to the years of schooling, men who had completed 12 or more years of schooling reported a lower prevalence of tobacco use, while it was the highest among those who never visited the school or had less than five years of schooling. Tobacco consumption was the highest (52% who consume any form of tobacco) among the men belonging to the scheduled tribes. Also, lower consumption of tobacco in any form (22%), cigarette smoking (11%), and bidi smoking (35%) was found among the men who have their place in the highest wealth quintiles. However, tobacco consumption was the highest among the men who belong to the lowest wealth quintile.

Table 4 presents the results of adjusted odds ratios from logistics regression for any tobacco use, smoking cigarette, and smoking bidi among men aged 15-49 years with selected background characteristics. Among the men aged 35-49 years, the odds were significantly higher for consuming any form of tobacco (AOR: 7.36, CI: 6.72-8.05), smoking cigarettes (AOR: 2.56, CI: 2.23-2.94), and smoking bidi (AOR: 7.12, CI: 4.75-8.82) compared with their counterparts. Moreover, men who live in urban areas are more likely to consume any form of tobacco, smoke cigarette, and smoke bidi compared with rural residents. Educational attainments (years of schooling) significantly affect tobacco use. Men with 12 or more years of schooling were significantly less likely to use tobacco, cigarettes, or bidi compared to illiterate and those with less than five years of schooling. Men from the scheduled tribes' caste were more likely to consume any form of tobacco (AOR: 1.15; CI: 1.04-1.26) than men from other caste categories. For the predictor wealth quintiles, it is observed that the probability of consuming any form of tobacco, smoking cigarette, and smoking bidi declines with the rising wealth status.

As presented in Table 5, the null model explained 15% of the clustering of tobacco use at the state level, while 29% and 38% of the clustering at the PSU and household levels, respectively. While considering the full model with a set of covariates as fixed effects, the clustering in tobacco use has increased significantly at the household level (48%). There has not been substantial changes at the PSU and household levels. Analyzing the fixed effects of various covariates, it is evident that men between the ages of 20-34 and 35-49 had greater adjusted odds ratios (AOR = 9.70, CI 8.9-10.5) for any tobacco use than those between 15 and 19 years. In terms of education, men with greater levels of education had lower chances (AOR: 0.18, CI: 0.16-0.19) than males without any education. Those from schedule caste and tribes had higher tobacco consumption as compared to those from non-SC/ST and non OBC (AOR: 1.40, CI: 1.30-1.50).

The percentage of men who made cessation efforts for quitting tobacco received advice from the healthcare provider and had exposure to secondhand smoke by selected background characteristics described in Table 6. Men who were in the age category 35-49 years attempted to stop consuming tobacco (30%). However, 58% of men who visited the hospital were advised to quit tobacco in the last 12 months, and 62% were exposed to secondhand smoke. There were not many differences in the percentage of cessation efforts made by men in rural and urban areas. Barely 25% of illiterate men tried to stop consuming tobacco in the last 12 months. Though 27% of men tried to quit tobacco in the last 12 months and 69% of men are exposed to secondhand smoke, 51% of men who received advice for quitting tobacco and visited the hospital in the last 12 months belong to the lowest wealth quintile.

Men aged 15-49 years who had tried to stop using any form of tobacco had been advised to quit tobacco and were exposed to secondhand smoke by states and union territories (UTs) in India, as shown in Table 7. Overall, 30% of men had tried to quit tobacco, 62% were exposed to secondhand smoke, and 54% of men who were advised to quit tobacco visited the hospital in the last 12 months. The highest percentage of men were reported for quitting tobacco in Manipur (48%), followed by Odisha (46%), Uttarakhand (45%), and Gujarat (41%). Most of the men who visited healthcare providers received advice to quit tobacco found in the states Punjab (82%), Haveli and Daman Diu (75%), and Andhra Pradesh (68%). Also, exposure to secondhand smoke among men was found to be the highest in Mizoram (91.5%), Delhi (81.4%), and Rajasthan (80%).

## 4. Discussion

The latest dataset from the National Family Health Survey-5 has been utilized to study the prevalence of tobacco consumption, types of tobacco use, and cessation of any form of tobacco consumption among men aged 15 and above years in India. With 275 million tobacco consumers, India is the second-largest tobacco consumer in the world after China [26]. However, there is a substantial decline in the prevalence of tobacco use among adults, but still, adequate socioeconomic inequalities are found in the prevalence of tobacco use among adults. The result from the study reveals that the overall prevalence of tobacco consumption (any form) was 38%, while the prevalence of any form of tobacco was 29% in rural and 43% in urban areas. Cigarette smoking and bidi smoking were more frequent than other tobacco products, such as paan masala or gutka, khaini, and bidis, in rural and urban areas. Bidi smoking and the use of khaini were more significant in rural areas than in urban areas. Men living in urban areas are more likely to consume tobacco, smoke cigarettes, and smoke bidi than rural residents. There were slight differences in the percentage of cessation efforts made by men in rural and urban areas. The prevalence of tobacco is the highest in the age category 50-64 years across rural and urban areas. For instance, rural-urban differentials in tobacco use have been documented well in the pieces of literature [7, 10, 27].

There is a subsequent reduction in tobacco use among adult men 15 years and above. However, it is still prevalent in many countries, and India is no exception. Relatively, death due to tobacco consumption (8 million deaths per year) appears to receive less attention globally. In 2012, a study conducted for 13 low-middle-income countries reported that 769 million are tobacco users [28]. In addition, another study estimated that 14 countries from the GATS survey highlighted 852 million are tobacco users, including 275 million in India [29]. A recent study has reported that tobacco consumption among men has risen by around 40 million. The estimates showed that tobacco consumption has increased from 1.050 billion in 2000 to 1.093 billion in 2018 [30]. India is more complex because of various smoking and smokeless tobacco products. Our study found that men who belong to the schedule tribe category and reside in rural areas had the highest prevalence of tobacco consumption. Tobacco consumption was the highest (52% who consume any form of tobacco) among the men belonging to the scheduled tribes. Men from the scheduled tribes' caste were more likely to consume tobacco than men from other caste categories. Compared with other countries, India has the second largest country in tobacco production after China, and many of these tobacco products are manufactured in the cottage and small industries in India [31].

Young age and peer pressure are the dominant risk factors for tobacco consumption. Among all tobacco consumption, smoking is persistent, where adolescence phase is a very potential stage to develop this habit. A study by Kamble et al. among 400 college students in Delhi found that 23% were ever smoked, 16% were current smokers, and most were adolescents [32]. The result from our study pointed out that the consumption of any form of tobacco and smoking five or more cigarettes/bidis were the highest in the 35-49 years of age group (51% and 57%, respectively) than the smokers of other age categories. Also, the odds were significantly higher for consuming any form of tobacco, smoking cigarettes, and smoking bidi compared with their counterparts. A study by Singh and Kashyap suggested that most tobacco users are young and mainly belong to the northeastern states [33]. In addition, initiation of tobacco consumption at an early age leads to mature and regular tobacco consumption at later ages and results in various severe health issues. Plaipudi et al. reported higher consumption of tobacco use found in the age group of 45 to 64 years [28].

Although the age at initiation and the age at quitting tobacco consumption have been interesting issues for policymakers [34], the cessation of tobacco consumption is the prime goal for many countries to control the tobacco epidemic. Further, it is well documented that aggressive tobacco cessation may result in several benefits to achieving the targets. In addition, tobacco cessation is important and strengthens the program to reduce tobacco consumption, which is integral to cultural practice [35]. Findings from our study expose that among all Indian states and union territories (UTs), tobacco consumption was the highest in northeastern states. Almost similar findings were found in a previous study [10]. Overall, 30% of men had tried to quit tobacco, 62% were exposed to secondhand smoke, and 54% of men were advised to quit tobacco when they visited the hospital in the last 12 months. However, it is a challenging and complex process to complete cessation of tobacco consumption among current users. Due to higher reversal rate in the process of behavior change where a person passes through stages of precontemplation, contemplation, preparation, action, and confirmation, an extraordinary effort is required to promote it and prevent the reversal. There may be many benefits of quitting tobacco before age 50, as they would reduce almost half of the risk of dying in the next 15 years [36]. Intensive promotion and preventive initiatives have been adopted internationally and nationally to combat the tobacco epidemic. In this arena, 181 countries came into a single platform where WHO (World Health Organization) organized a framework for tobacco control (FCTC) [37]. Reduction in smoking cigarettes and smokeless tobacco remained the main priority of the convention. The government of India is also not behind in implementing such policies, making lots of effort to control the current situation where healthcare providers are key drivers. In 2003, the Indian government created a Cigarettes and Other Tobacco Products Act (COTPA) to reduce tobacco use through taxation, pictorial depiction of tobacco products, and other measures [22]. A recent study reported that 35% of tobacco users were in 2009-10, reduced to 27% in 2016-17 [38]. Reduction in tobacco use may result from measures adopted by India's government.

This study identified that men from the lowest wealth quintile had the highest prevalence of tobacco consumption, while it was the lowest among men belonging to the highest wealth quintile. Tobacco consumption was the highest among the men who belong to the lowest wealth quintile. The chance of consuming any form of tobacco, smoking cigarette, and smoking bidi declines with the rising wealth status. Twenty-seven percent of men tried to quit tobacco in the last 12 months, 69% of men are exposed to secondhand smoke, and 51% of men who received advice for quitting tobacco belongs to the lowest wealth quintile. This may be due to the better advantages of health education on the upper wealth quintile than lower wealth quintile [39]. Contextual effects can explain the higher prevalence of smoking in urban areas. The “contextual effect” emphasizes the significance of the social and demographic composition of people's lives in urban areas [40]. Some previous studies found that higher education leads to lower tobacco consumption [41–43].

Educational attainments (years of schooling) significantly affect tobacco use. The prevalence shows a declining trend as the level of schooling increases in rural and urban areas. According to the years of schooling, men who had completed 12 or more years reported a lower prevalence of tobacco use, while it was the highest among those who never visited school or had less than five years of schooling. Men with 12 or more years of schooling were significantly less likely to use tobacco, cigarettes, or bidi compared to illiterate and those with less than five years of schooling. Around twenty-five percent of illiterate men tried to stop consuming tobacco in the last 12 months.

Moreover, many studies found that tobacco consumption among the Muslim population is lower than any other religion [5, 44, 45]. In contrast, this study has found that Muslim adults consume more tobacco than other religions, as one study found similar findings [46]. Furthermore, out of 36,378 current tobacco users, thirty percent intend to quit tobacco in the 15-49 age group. Looking for the predictors of quitting tobacco, adults who reside in rural areas had greater intention to quit tobacco which is a consistent finding from previous studies [34, 47, 48]. Secondhand smoke (SHS) is always considered a public health challenge in India, as there is no doubt that the risk of secondhand smoke exposure in India is relatively high. From previous GATS I and II studies, proportion of SHS was reported 48% and 35% in 2009-10 and 2016-17, respectively [49]. In contrast, this study has found that more than 60% of adults aged 15-49 had been exposed to SHS. Since the COTPA act and MPOWER strategies, tobacco use has declined significantly. However, tobacco consumption remains a burning issue due to the poor implementation and dissemination of law and policy [50].

Analysis of clustering in an event or behavior at different levels provides opportunities for suitable intervention for changing discourse of the events/behaviors. The clustering in tobacco use/smoking among men at different levels is another significant contribution of this study. Results of multilevel models indicated that, across all levels, tobacco use at the household level was found to be more concentrated (48%), which may be primarily explained by cognitive factors explaining intergenerational behavioral transition. Also, a substantial clustering in tobacco use at community/PSU level (29%) may be resulted due to neighborhood and other contextual effects, especially exhibited in negative behaviors. Last but not the least, a 13% clustering effect at the state level indicated the role of cultural and ecological factors affecting tobacco use/smoking in India.

## 5. Conclusions and Recommendations

Overall, the study concluded that predictors such as age, educational status, place of residence, caste, and wealth quintile had a statistically significant association with tobacco use in any form. Cessation attempt to stop tobacco smoking is a result of contribution by healthcare providers who adviced to quit tobacco to those individulas who visited to avail healthcare services. Our study also exposes that tobacco consumption was the highest in northeastern states among all Indian states and union territories (UTs). Nonetheless, the current scenario still warrants more significant public health attention and demand for better policy implementation. The study recommends three prone strategies to address the issue of tobacco epidemic in the country. First is emphasizing at specific population segments rather than adopting universal approach of prevention and control. This is primarily due to the fact that tobacco uses are higher among the northeastern states, scheduled caste and tribe, and lowest wealth quintile. Consequently, there is a need to focus on state-level approaches to control the tobacco epidemic. In addition, adults who belong to scheduled castes, tribes, and the lowest wealth quintile need to adopt a target-based approach to control tobacco use. Second, there is a need to adopt suitable strategies to focus at awareness and capacity building of rural folk as most of COTPA and MPOWER strategies are primarily targeting urban clients. The study also found that tobacco use is much higher in rural areas than in urban areas; hence, the study demanded the promotion of awareness regarding the adverse effect of tobacco use, especially in rural areas. Third, the health system response to the tobacco epidemic in the country should be strengthened by training of service providers in promoting cessation efforts through suitable counselling of all the patients visiting them in the context of tobacco usage in any form. The study also demands to educate individuals about the harmful effects of tobacco usage which is the key driver of increasing burden of NCDs, through community-based organizations.

## 6. Limitations of the Study

The study has some limitations too. The first limitation of this study is that tobacco use is consistently underreported in Indian scenarios. The second limitation of this is that the study is based on cross-sectional data, so it can neither explain the whole scenario of the tobacco epidemic nor could it draw causal inferences based on study findings. Moreover, the study only analyzed the male data. Hence, it could not give a picture of gender differences in tobacco use; this could also be one limitation. However, apart from all the limitations, the study provides a broad picture of the tobacco epidemic among adult men in India.

## Figures and Tables

**Table 1:** Percentage of the population age 15 years and over who currently use any tobacco, according to background characteristics, India, 2019-21.

Background characteristic	Urban	Rural	Total	Number of men
Age				
15-19	4.24	7.6	6.5	125,345
20-34	22.97	33.59	29.9	316,520
35-49	38.58	54.62	48.9	254,932
50 or above	36.57	57.87	50.9	291,917
Level of schooling				
No schooling	53.8	65.0	62.7	156,482
Primary	48.3	59.0	56.0	137,823
Secondary	29.0	36.0	33.4	521,487
Higher	14.0	17.0	15.2	172,272
Religion of household head				
Hindu	29.0	43.5	38.8	808,894
Muslim	31.6	43.1	38.0	124,581
Others	19.7	30.4	26.5	55,388
Caste/tribe of household head				
Unreserved	25.5	37.7	32.1	222,800
OBC	27.0	40.3	35.8	414,322
SC/ST	35.4	48.1	44.9	302,693
Wealth quintile				
Lowest	58.5	58.3	28.3	171,071
Second	46.3	47.4	47.3	188,773
Middle	39.8	38.3	38.7	201,517
Fourth	31.2	30.1	30.9	208,657
Highest	19.9	22.2	20.5	218,844
Total	**28.8**	**42.7**	**38.0**	**988,713**

**Table 2:** Percentage of men aged 15-49 and men age 15-54 by their use of tobacco and percent distribution of those who smoke cigarettes or bidis by number of cigarettes/bidis smoked each day on average, India, 2019-21.

Tobacco use	Urban	Rural	Total 15-49	*p* value (rural and urban)	Total 15-54
Use of tobacco					
Smokes cigarettes	14.6	12.5	13.2	0.000	13.3
Smokes *bidis*	4.5	8.3	7	0.000	7.8
Smokes cigars	0.6	0.5	0.6	0.716	0.6
Smokes a pipe	0.1	0.1	0.1	0.611	0.1
Smokes a *hookah*	0.3	0.7	0.5	0.000	0.6
Chews *paan masala* or *gutka*	12	15.8	14.5	0.000	14.2
Uses *khaini*	6.6	14.5	11.7	0.000	12.1
Chews *paan* with tobacco	3.4	6	5.1	0.000	5.3
Other chewing tobacco	1.5	1.9	1.8	0.000	1.8
Uses snuff	0	0.1	0.1	0.022	0.1
Other	0.3	0.6	0.5	0.000	0.5
Does not use tobacco	67.9	57.1	60.9	0.000	59.7
Number of respondents	32,852	60,291	93,144		1,01,839
Number of cigarettes smoked each day on average					
<5	67.8	74.4	71.8	0.000	70.9
5-9	17.9	10.5	13.4		13.8
10-14	7.1	5.8	6.3		6.6
15-24	1.5	1.4	1.4		1.5
25 or more	1.3	0.4	0.7		0.8
Missing	4.4	7.5	6.3		6.4
Number of cigarette smokers	4784	7537	12,321		13,513
Number of bidis smoked each day on average					
<5	19.4	20.8	20.5	0.000	19.9
5-9	24.4	25.9	25.6		25.9
10-14	33.9	27.1	28.6		28.1
15-24	14.7	20.7	19.3		19.8
25 or more	3.4	4.2	4		4.4
Missing	4.1	1.3	1.9		1.8
Number of *bidis* smokers	1474	5007	6482		7974
Number of cigarettes/bidis smoked each day on average					
<5	55.7	50.2	52.1	0.000	49.5
5-9	20	19.2	19.4		20.1
10-14	13.7	15.5	14.9		15.6
15-24	6.3	10.3	9		9.8
25 or more	2.3	3	2.8		3.1
Missing	1.9	1.8	1.9		1.9
Number of cigarette/*bidis* smokers	5633	11,113	16,746		19,066

**Table 3:** Percentage of men aged 15-49 who use any kind of tobacco and percentage who smoke cigarettes or bidis and, among those who smoke cigarettes or bidis, the percentage who smoke 5 or more cigarettes or bidis each day on average, by background characteristics, India, 2019-21.

Background characteristic	Percentage who use any kind of tobacco		Percentage who smoke cigarettes		Percentage who smoke *bidis*		Number of men	Percentage who smoke 5 or more cigarettes/*bidis* each day on average		Number of men who smoke cigarettes/*bidis*
	%	CI at 95%	%	CI at 95%	%	CI at 95%		%	CI at 95%	
Age										
15-19	14.6	13.7-15.5	6.2	5.5-6.9	2.5	2.0-3.0	16,407	17.8	15.7-20.1	1184
20-34	39.1	38.2-39.9	14.9	14.3-15.6	7.1	6.7-7.6	41,743	36.1	35.0-37.1	7776
35-49	51.8	50.9-52.8	14.5	13.8-15.2	15.4	14.716.1	35,118	57.1	56.0-58.1	8777
Residence										
Urban	32.4	31.3-33.6	14.6	13.7-15.5	6.1	5.5-6.7	32,896	42.5	41.2-43.8	5813
Rural	43.5	42.8-44.1	12.5	12.1-12.9	11.3	10.8-11.7	60,371	46.6	45.7-47.5	11,924
Schooling										
No schooling	62.2	60.5-63.8	15.2	13.9-16.5	22.6	21.2-24.1	9760	61.9	60.1-63.6	3081
Primary	63.1	61.4-64.8	16.9	15.6-18.2	20.7	19.3-22.2	10,782	55.1	53.4-56.7	3364
Secondary	37.3	36.6-38.0	12.8	12.3-13.4	7.3	6.9-7.7	54,223	40.2	39.2-41.2	9077
Higher	20.5	19.3-21.7	11.3	10.3-12.4	2.0	1.7-2.4	18,503	27.8	26.0-29.7	2214
Religion										
Hindu	39.6	38.9-40.2	12.7	12.2-13.2	9.0	8.7-9.4	73,730	44.1	43.3-44.9	13,471
Muslim	41.8	40.3-43.3	14.4	13.3-15.5	12.1	11.0-13.2	14,653	49.8	48.1-51.6	3282
Others	33.1	30.8-35.5	17.5	15.6-19.6	7.6	6.7-8.6	4885	45.4	42.3-48.5	984
Caste/tribe										
Unreserved	35.0	33.7-36.3	12.6	11.6-13.7	7.8	7.0-8.6	19,915	49.2	47.5-50.9	3314
OBC	35.7	34.8-36.5	10.9	10.3-11.4	6.5	6.1-6.9	40,394	39.2	38.0-40.5	5876
SC/ST	46.8	45.8-47.8	15.1	14.3-15.9	13.2	12.5-13.9	28,408	49.6	48.4-50.9	6350
Wealth quintile										
Lowest	59.7	58.5-61.0	14.8	13.9-15.7	19.2	18.1-20.3	15,627	49.1	47.6-50.6	4352
Second	49.4	48.2-50.6	14.6	13.7-15.6	13.5	12.6-14.4	18,521	47.4	45.9-48.9	4239
Middle	38.9	37.7-40.0	12.8	12.0-13.6	8.5	7.9-9.3	19,855	47.3	45.6-48.9	3533
Fourth	31.8	30.6-33.0	12.8	11.9-13.7	5.2	4.7-5.8	20,686	42.4	40.7-44.1	3213
Highest	22.3	20.9-23.6	11.5	10.4-12.7	2.9	2.4-3.4	18,578	35.1	33.2-37.1	2401
Total age 15-49	**39.6**	**39.6-39.6**	**13.2**	**13.2-13.2**	**9.4**	**9.4-9.4**	**93,267**	**45.2**	**45.5-46.0**	**17,737**
Age 50-54	**54.0**	**53.9-54.0**	**13.7**	**13.7-13.7**	**20.5**	**20.5-20.5**	**8572**	**64.6**	**62.7-66.5**	**2462**
Total age 15-54	**40.7**	**40.8-40.8**	**13.3**	**13.3-13.3**	**10.4**	**10.4-10.4**	**1,01,839**	**47.6**	**46.9-48.3**	**20,198**

**Table 4:** Unadjusted and adjusted effect of any tobacco use, smoking cigarette, and smoking bidi by background characteristics among 15-49 years of the men in India.

	Any tobacco use				Smoking cigarette				Smoking bidi			
	Unadjusted odds ratio	CI at 95%	Adjusted odds ratio	CI at 95%	Unadjusted odds ratio	CI at 95%	Adjusted odds ratio	CI at 95%	Unadjusted odds ratio	CI at 95%	Adjusted odds ratio	CI at 95%
Age												
15-19 (ref)												
20-34	3.85^∗∗∗^	3.68-4.03	4.97^∗∗∗^	4.73-5.22	2.94^∗∗∗^	2.74-3.15	3.16^∗∗∗^	2.94-3.4	3.87^∗∗∗^	3.47-4.32	4.37^∗∗∗^	3.89-4.9
35-49	6.43^∗∗∗^	6.14-6.74	7.38^∗∗∗^	7.02-7.77	2.96^∗∗∗^	2.77-3.18	3.11^∗∗∗^	2.89-3.35	9.31^∗∗∗^	8.36-10.37	8.88^∗∗∗^	7.93-9.95
Residence												
Urban (ref)												
Rural	1.48^∗∗∗^	1.44-1.53	0.84^∗∗∗^	0.81-0.88	0.90^∗∗∗^	0.86-0.93	0.72^∗∗∗^	0.68-0.75	1.79^∗∗∗^	1.89-0.02	1.07^∗∗^	1-1.14
Schooling												
No schooling (ref)												
Primary	1.02	0.97-1.08	1.15^∗∗∗^	1.08-1.22	1.15^∗∗∗^	1.07-1.24	1.18^∗∗∗^	1.09-1.27	0.89^∗∗∗^	0.83-0.95	0.99	0.92-1.07
Secondary	0.39^∗∗∗^	0.38-0.41	0.72^∗∗∗^	0.68-0.75	0.90^∗∗∗^	0.85-0.95	1.11^∗∗∗^	1.04-1.18	0.31^∗∗∗^	0.30-0.33	0.53^∗∗∗^	0.5-0.57
Higher	0.18^∗∗∗^	0.17-0.19	0.34^∗∗∗^	0.32-0.36	0.73^∗∗∗^	0.68-0.79	0.83^∗∗∗^	0.76-0.9	0.09^∗∗∗^	0.08-0.10	0.16^∗∗∗^	0.14-0.18
Religion												
Hindu (ref)												
Muslim	1.00	0.96-1.04	1.07^∗∗^	1.02-1.13	1.35^∗∗∗^	1.27-1.42	1.38^∗∗∗^	1.3-1.48	1.02	0.95-1.09	1.05	0.96-1.14
Others	1.23^∗∗∗^	1.18-1.28	1.12^∗∗∗^	1.07-1.17	2.82^∗∗∗^	1.27-2.96	2.54^∗∗∗^	2.41-2.67	1.58^∗∗∗^	1.49-1.67	1.41^∗∗∗^	1.32-1.51
Caste/tribe												
Unreserved (ref)												
OBC	1.19^∗∗∗^	1.15-1.24	0.97	0.93-1.01	0.79^∗∗∗^	0.75-0.83	0.81^∗∗∗^	0.76-0.86	1.10^∗∗∗^	1.03-1.18	0.89^∗∗∗^	0.83-0.96
SC/ST	1.84^∗∗∗^	1.77-1.91	1.22^∗∗∗^	1.16-1.27	1.49^∗∗∗^	1.41-1.57	1.19^∗∗∗^	1.12-1.26	2.02^∗∗∗^	1.90-2.16	1.28^∗∗∗^	1.19-1.38
Wealth quintile												
Lowest (ref)												
Second	0.68^∗∗∗^	0.66-0.71	0.73^∗∗∗^	0.7-0.76	1.08^∗∗^	1.02-1.14	1.08^∗∗^	1.02-1.15	0.76^∗∗∗^	0.72-0.80	0.93^∗∗^	0.87-0.98
Middle	0.48^∗∗∗^	0.46-0.50	0.51^∗∗∗^	0.49-0.53	0.98	0.93-1.04	0.99	0.93-1.05	0.51^∗∗∗^	0.48-0.54	0.70^∗∗∗^	0.66-0.75
Fourth	0.35^∗∗∗^	0.33-0.36	0.38^∗∗∗^	0.36-0.4	0.88^∗∗∗^	0.83-0.93	0.86^∗∗∗^	0.8-0.92	0.35^∗∗∗^	0.32-0.37	0.57^∗∗∗^	0.52-0.61
Highest	0.21^∗∗∗^	0.20-0.22	0.26^∗∗∗^	0.24-0.27	0.71^∗∗∗^	0.67-0.76	0.67^∗∗∗^	0.61-0.72	0.22^∗∗∗^	0.20-0.24	0.44^∗∗∗^	0.39-0.48

^∗∗∗^Significant at *p* value ≤ 0.005, and ^∗∗^significant at *p* value ≤ 0.05.

**(a):** 

	Adjusted odds ratio (AOR)	95% confidence interval	
		Lower limit	Upper limit
Age			
15-19 (ref)			
20-34	9.70^∗∗∗^	8.9	10.47
35-49	18.0^∗∗∗^	16.56	19.65
Residence			
Urban (ref)			
Rural	0.88^∗∗∗^	0.82	0.95
Schooling			
No schooling (ref)			
Primary	1.00	0.95	1.13
Secondary	0.54^∗∗∗^	0.5	0.58
Higher	0.18^∗∗∗^	0.16	0.19
Religion			
Hindu (ref)			
Muslim	1.08	0.99	1.19
Others	0.80^∗∗∗^	0.71	0.89
Caste/tribe			
Unreserved (ref)			
OBC	1.06	0.99	1.13
SC/ST	1.40^∗∗∗^	1.3	1.5
Wealth quintile			
Lowest (ref)			
Second	0.73^∗∗∗^	0.69	0.78
Middle	0.55^∗∗∗^	0.51	0.6
Fourth	0.41^∗∗∗^	0.38	0.49
Highest	0.28^∗∗∗^	0.25	0.31

**(b):** 

	Variance	Intraclass correlation coefficient (ICC)
Random effect		
*State*	**0.78**	**15%**
*PSU*	**0.76**	**29%**
*Household*	**0.48**	**38%**
Mixed Effect		
*State*	**0.80**	**13%**
*PSU*	**1.07**	**29%**
*Household*	**1.19**	**48%**

^∗∗∗^Significant at *p* value ≤ 0.005.

**Table 6:** Percentage of men aged 15-49 who tried to stop tobacco, who were advised to quit tobacco, who visited a doctor, and who were exposed to secondhand smoke, by background characters, India, 2019-21.

	Tried to quit tobacco in the last 12 months		Number of current users of tobacco	Advised to quit any form of tobacco		Number of current tobacco users who visited to healthcare provider	Secondhand smoke (SHS)		Number of men
	%	CI at 95%		%	CI at 95%		%	CI at 95%	
Age									
15-19	25.6	22.9-28.4	2554	36.5	28.1-45.7	333	58.5	57.1-59.8	16,385
20-34	30.8	29.6-32.1	17,382	50.9	47.5-54.2	2725	63.4	62.6-64.3	41,688
35-49	30	28.9-31.2	19,416	58.1	55.0-61.2	3076	62.3	61.3-63.2	35,071
Residence									
Urban	28.4	26.5-30.3	11,378	57.7	52.5-62.9	1578	60.5	59.3-61.7	32,852
Rural	30.8	29.9-31.7	27,974	52.3	50.0-54.7	4556	63	62.4-63.6	60,291
Schooling									
No schooling	25.2	23.4-27.1	6473	55.9	50.6-61.1	909	65.4	63.8-67.0	9747
Primary	28.1	26.3-30.0	7258	54	48.7-59.1	1117	68.8	67.1-70.3	10,768
Secondary	32.1	31.0-33.2	21,577	53.6	50.7-56.5	3410	61.7	60.9-62.4	54,151
Higher	30.8	27.9-34.0	4044	51	43.3-58.7	698	57.9	56.3-59.3	18,478
Religion									
Hindu	30.8	29.8-31.7	31,094	54.6	52.2-57.0	4723	62.1	61.4-62.7	73,632
Muslim	25.9	23.9-28.1	6534	50.8	44.8-56.8	1172	62.9	61.4-64.4	14,633
Others	34.1	29.9-38.6	1724	49.7	40.0-59.3	238	60.3	57.8-62.7	4878
Caste/tribe									
Unreserved	32.6	30.4-34.9	7524	58.9	53.2-64.5	1178	62.7	61.3-64.0	19,221
OBC	31.9	30.6-33.2	15,551	52.6	49.1-56.1	2243	61	60.1-61.9	38,986
SC/ST	28.8	27.6-30.1	14,350	51.1	47.6-54.5	2088	65.6	64.6-66.6	27,418
Wealth quintile									
Lowest	27.1	25.7-28.6	9956	51.1	47.0-55.1	1540	68.5	67.3-69.7	15,606
Second	29.3	27.8-30.9	9750	51.2	47.1-55.3	1486	64.2	63.1-65.4	18,497
Middle	32	30.3-33.7	8227	55.4	50.7-60.0	1339	60.3	59.2-61.5	19,829
Fourth	32.2	30.1-34.5	7012	56.2	50.4-61.8	1076	60.6	59.3-61.9	20,658
Highest	31.7	28.6-35.0	4408	57.9	49.8-65.6	692	58.2	56.6-59.8	18,553
Total age 15-49	**30.1**	**30.1-30.1**	**39,233**	**53.7**	**51.5-55.9**	**6134**	**62.1**	**61.5-62.7**	**93,144**
Age 50-54	**29.1**	**29.1-29.1**	**4994**	**66.2**	**60.4-71.5**	**949**	**60.4**	**58.5-62.3**	**8695**
Total age 15-54	**30**	**30.0-30.0**	**44,227**	**55.4**	**53.3-57.4**	**7083**	**62**	**61.4-62.5**	**1,01,839**

**Table 7:** Among men age 15-49 who currently use any kind of tobacco, percentage who have tried to stop using any tobacco and, among current users of tobacco who visited a doctor or other healthcare provider in the 12 months preceding the survey, percentage who were advised to quit any form of tobacco and percentage who were exposed to secondhand smoke.

	Tried to quit tobacco in the last 12 months		Advised to quit any form of tobacco who visited healthcare provider in the last 12 months		SHS (secondhand smoke)	
	%	CI at 95%	%	CI at 95%	%	CI at 95%
Andaman & Nicobar Islands	29.1	21.5-38.2	51.8	29.1-73.7	62.7	56.5-68.6
Andhra Pradesh	34.0	28.5-40	68.2	55.5-78.7	62.7	59.9-65.5
Arunachal Pradesh	18.8	16.4-21.4	54.6	45.5-63.4	58.2	55.8-60.4
Assam	11.9	10.6-13.5	36.7	30.9-42.8	46.1	44.5-47.8
Bihar	31.2	28.9-33.5	50.8	43-58.5	70.3	68.8-71.9
Chandigarh	37.3	19.4-59.5	0	0-0	68.4	58.5-76.9
Chhattisgarh	21.7	19.3-24.4	46.7	37.4-56.2	68.2	66.4-70.0
Goa	18.1	10.1-30.3	68.6	22.2-94.4	51.6	45.4-57.7
Gujarat	40.6	38.2-43.1	56.8	48.9-64.4	69.8	68.1-71.4
Haryana	22.2	19.3-25.5	52.6	42.5-62.4	73.6	71.9-75.3
Himachal Pradesh	30.6	25.5-36.3	66.6	44.3-83.4	75.3	72.4-78.1
Jammu & Kashmir	11.8	9.5-14.4	38.5	31.6-45.9	65.3	63.2-67.4
Jharkhand	25.2	22.8-27.7	42.3	34.8-50.3	69.0	67.2-70.8
Karnataka	27.6	24.5-30.9	56.2	48.2-63.9	37.6	35.6-39.7
Kerala	35.4	29.0-42.5	52.6	38.4-66.4	55.2	52.2-58.2
Ladakh	3.5	1.1-10.3	15.1	5.7-34.2	59.6	53.7-65.3
Lakshadweep	8.6	3.2-21.2	0.0	0-0	38.1	29.8-47.1
Madhya Pradesh	33.9	31.8-36.1	57.8	52-63.4	75.1	73.8-76.4
Maharashtra	36.2	32.8-39.6	62.9	53.6-71.4	54.3	52.1-56.4
Manipur	47.6	43.2-52.1	29.7	18.6-43.7	76.5	73.1-79.7
Meghalaya	33.7	29.6-38.1	57.3	46.2-67.7	53.8	50.6-57.1
Mizoram	28.3	23.7-33.5	40.6	28.8-53.5	91.5	88.6-93.8
Nagaland	19.3	15.9-23.3	16.0	7.6-30.8	71.7	68.6-74.6
Nagar Haveli And Daman & Diu	38.4	29.6-48	74.8	45.6-91.3	64.8	58.7-70.4
NCT of Delhi	35.4	31-40.1	65.8	53.7-76.1	81.4	79.2-83.3
Odisha	46.1	43.5-48.7	35.1	31.0-39.5	62.1	60.3-64.0
Puducherry	28.2	11.8-53.6	61.9	24.5-89.1	58.8	50.2-66.8
Punjab	33.8	28.5-39.4	82.3	69.6-90.4	53.8	51.6-56.1
Rajasthan	23.4	21.6-25.3	41.6	37.3-46.1	80.1	79-81.3
Sikkim	18.8	11.8-28.5	42.3	19.2-69.3	50.3	42.6-58
Tamil Nadu	37.1	32.5-41.9	65.9	55.2-75.3	43.7	41.4-46
Telangana	39.1	33.4-45	65.5	54.2-75.4	54.5	52.0-57.0
Tripura	13.9	11.1-17.3	55.2	46.4-63.6	76.9	73.5-80.0
Uttar Pradesh	32.3	30.8-33.8	50.0	46.4-53.5	77.8	76.8-78.6
Uttarakhand	44.0	38.1-50.1	30.0	19.6-43.1	85.8	83.1-88.1
West Bengal	22.7	20.5-25.1	55.1	49.0-61.2	68.1	66.1-70

## Data Availability

The data is freely available from gateway to demographic and health survey (DHS) and can be obtained from the following link: https://dhsprogram.com/data/available-datasets.cfm.

## References

[1] Tselengidis A., Dance S., Adams S., Freeman B., Cranwell J. (2023). Tobacco advertising, promotion, and sponsorship ban adoption: a pilot study of the reporting challenges faced by low- and middle-income countries. *Tobacco Induced Diseases*.

[2] Rezaei N., Farzadfar F. (2020). Points to consider regarding tobacco hindrance. *Archives of Iranian Medicine*.

[3] Smith E. A., Malone R. E. (2020). An argument for phasing out sales of cigarettes. *Tobacco Control*.

[4] John R. M., Sinha P., Munish V. G., Tullu F. T. (2021). Economic costs of diseases and deaths attributable to tobacco use in India, 2017–2018. *Nicotine & Tobacco Research*.

[5] Islam M. S., Saif-Ur-Rahman K. M., Bulbul M. M. I., Singh D. (2020). Prevalence and factors associated with tobacco use among men in India: findings from a nationally representative data. *Environmental Health and Preventive Medicine*.

[6] Sivapuram M. S., Nagarathna R., Anand A., Patil S., Singh A., Nagendra H. R. (2020). Prevalence of alcohol and tobacco use in india and implications for COVID-19 - Niyantrita Madhumeha Bharata study projections. *Journal of Medicine and Life*.

[7] Shahbabu B., Dasgupta A., Sarkar I., Sarkar K. (2020). Rural-urban differentials in predicting tobacco consumption pattern among males above 15 years: a cross-sectional community survey. *Medical Journal of Dr. DY Patil Vidyapeeth*.

[8] Ng M., Freeman M. K., Fleming T. D. (2014). Smoking prevalence and cigarette consumption in 187 countries, 1980-2012. *JAMA*.

[9] Chen D. T. H., Millett C., Filippidis F. T. (2021). Prevalence and determinants of dual and poly-tobacco use among males in 19 low-and middle-income countries: implications for a comprehensive tobacco control regulation. *Preventive Medicine*.

[10] Gupta V., Yadav K., Anand K. (2010). Patterns of tobacco use across rural, urban, and urban-slum populations in a north Indian community. *Indian journal of community medicine: official publication of Indian Association of Preventive & Social Medicine*.

[11] Agunwamba A. A., Kawachi I., Williams D. R., Finney Rutten L. J., Wilson P. M., Viswanath K. (2017). Mental health, racial discrimination, and tobacco use differences across rural-urban California. *The Journal of Rural Health*.

[12] Roberts M. E., Doogan N. J., Kurti A. N. (2016). Rural tobacco use across the United States: how rural and urban areas differ, broken down by census regions and divisions. *Health & Place*.

[13] Singh A., Ladusingh L. (2014). Prevalence and determinants of tobacco use in India: evidence from recent Global Adult Tobacco Survey data. *PLoS One*.

[14] Foulds J., Gandhi K. K., Steinberg M. B. (2006). Factors associated with quitting smoking at a tobacco dependence treatment clinic. *American Journal of Health Behavior*.

[15] Joshi U., Modi B., Yadav S. (2010). A study on prevalence of chewing form of tobacco and existing quitting patterns in urban population of Jamnagar, Gujarat. *Indian journal of community medicine: official publication of Indian Association of Preventive & Social Medicine*.

[16] Borland R., Partos T. R., Yong H. H., Cummings K. M., Hyland A. (2012). How much unsuccessful quitting activity is going on among adult smokers? Data from the International Tobacco Control Four Country cohort survey. *Addiction*.

[17] Chung-Hall J., Craig L., Gravely S., Sansone N., Fong G. T. (2019). Impact of the WHO FCTC over the first decade: a global evidence review prepared for the Impact Assessment Expert Group. *Tobacco Control*.

[18] World Health Organization (2011). *WHO report on the global tobacco epidemic, 2011: warning about the dangers of tobacco: executive summary (No. WHO/NMH/TFI/11.3)*.

[19] Song Y., Zhao L., Palipudi K. M. (2016). Tracking MPOWER in 14 countries: results from the Global Adult Tobacco Survey, 2008–2010. *Global Health Promotion*.

[20] Ruhil R. (2018). India has reached on the descending limb of tobacco epidemic. *Indian Journal of Community Medicine*.

[21] Dhot P. S. (2005). Amendments to Indian drugs and cosmetics act and rules pertaining to blood banks in armed forces. *Medical Journal, Armed Forces India*.

[22] Kaur J., Jain D. C. (2011). Tobacco control policies in India: implementation and challenges. *Indian Journal of Public Health*.

[23] Chaudhary A., Thakur A., Chauhan T. (2019). Creation of a smoke-free environment for children: an assessment of compliance to COTPA 2003 legislation in an urban area. *Indian Pediatrics*.

[24] Barik A., Rai R. K., Gorain A., Majumdar S., Chowdhury A. (2016). Socio-economic disparities in tobacco consumption in rural India: evidence from a health and demographic surveillance system. *Perspectives in Public Health*.

[25] Rabe-Hesketh S., Skrondal A. Understanding variability in multilevel models for categorical responses.

[26] Hasan M. M., Quazi A., Sarangapani N., Alam K. (2023). Age-specific prevalence and predictors of tobacco consumption among male adults in India: subnational inequality and associated risk factors. *Journal of Public Health*.

[27] Ozga J. E., Romm K. F., Turiano N. A. (2021). Cumulative disadvantage as a framework for understanding rural tobacco use disparities. *Experimental and Clinical Psychopharmacology*.

[28] Palipudi K. M., Gupta P. C., Sinha D. N. (2012). Social determinants of health and tobacco use in thirteen low and middle income countries: evidence from Global Adult Tobacco Survey. *PLoS One*.

[29] Giovino G. A., Mirza S. A., Samet J. M. (2012). Tobacco use in 3 billion individuals from 16 countries: an analysis of nationally representative cross-sectional household surveys. *The Lancet*.

[30] Tripathy J. P. (2020). Secondhand smoke exposure at home and public places among smokers and non-smokers in India: findings from the Global Adult Tobacco Survey 2016–17. *Environmental Science and Pollution Research*.

[31] Gupta P. C., Ray C. S. (2004). Epidemiology of betel quid usage. *Annals Academy of Medicine Singapore*.

[32] Kamble B. D., Acharya B. P., Jethani S., Chellaiyan V. G., Singh S. K., Chaku S. (2022). Tobacco smoking habits and nicotine dependence among the college students of University of Delhi, India. *Journal of Family Medicine and Primary Care*.

[33] Singh S. K., Kashyap G. C. (2016). Progression in tobacco use in India: an application of survival function analysis. *International Journal of Social Sciences and Management*.

[34] Kumar S., Patel R., Chauhan S., Gupte S. S. (2022). Prevalence, pattern, and cessation of tobacco consumption among older adults in India. *Journal of Substance Use*.

[35] Slama K. (2004). Current challenges in tobacco control. *The International Journal of Tuberculosis and Lung Disease*.

[36] Murthy P., Saddichha S. (2010). Tobacco cessation services in India: recent developments and the need for expansion. *Indian Journal of Cancer*.

[37] Yadav A., Singh P. K., Yadav N. (2020). Smokeless tobacco control in India: policy review and lessons for high-burden countries. *BMJ Global Health*.

[38] Tata Institute of Social Sciences (TISS), Mumbai and Ministry of Health and Family Welfare, Government of India (2018). *Global adult tobacco survey GATS 2 India 2016-17*.

[39] Prinja S., Kumar R. (2009). Reducing health inequities in a generation: a dream or reality?. *Bulletin of the World Health Organization*.

[40] Diez Roux A. V. (2001). Investigating neighborhood and area effects on health. *American Journal of Public Health*.

[41] Singh S. K., Schensul J. J., Kashyap G. C. (2017). The reach of media to smokers and smokeless tobacco users in India: evidence from the Global Adult Tobacco Survey (GATS). *Journal of Population and Social Studies*.

[42] Prabhakar B., Narake S. S., Pednekar M. S. (2012). Social disparities in tobacco use in India: the roles of occupation, education and gender. *Indian Journal of Cancer*.

[43] Biswas S., Syiemlieh J., Nongrum R., Sharma S., Siddiqi M. (2021). Prevalence of tobacco use in young adult literate girls of 18-25 years in Meghalaya, India: a cross-sectional study. *Asian Pacific Journal of Cancer Prevention*.

[44] Pasupuleti S. S., Mohan P., Babu P. J. (2021). Prevalence and predictors of tobacco use among currently married pregnant women in India. *Population Medicine*.

[45] Patel R., Kumar P., Srivastava S., Chauhan S. (2021). Change in socio-economic inequality of tobacco consumption among men in India: evidence from National Family Health Survey 2005-06 to 2015-16. *Journal of Substance Use*.

[46] Rani M., Bonu S., Jha P., Nguyen S. N., Jamjoum L. (2003). Tobacco use in India: prevalence and predictors of smoking and chewing in a national cross sectional household survey. *Tobacco Control*.

[47] Sarkar B. K., Arora M., Gupta V. K., Reddy K. S. (2013). Determinants of tobacco cessation behaviour among smokers and smokeless tobacco users in the states of Gujarat and Andhra Pradesh, India. *Asian Pacific Journal of Cancer Prevention*.

[48] Panda R., Venkatesan S., Persai D., Trivedi M., Mathur M. R. (2014). Factors determining intention to quit tobacco: exploring patient responses visiting public health facilities in India. *Tobacco Induced Diseases*.

[49] Verma M., Kathirvel S., Das M., Aggarwal R., Goel S. (2020). Trends and patterns of second-hand smoke exposure amongst the non-smokers in India-a secondary data analysis from the Global Adult Tobacco Survey (GATS) I & II. *PLoS One*.

[50] Venkatesan R., Jhamtani R. C., Gupta S., Vinchurkar S., Jain N. (2020). Smokeless tobacco dependence and cessation measures in India. *International Journal of Aging Research*.

